# Hierarchical HZSM-5 for Catalytic Cracking of Oleic Acid to Biofuels

**DOI:** 10.3390/nano11030747

**Published:** 2021-03-16

**Authors:** Mahashanon Arumugam, Chee Keong Goh, Zulkarnain Zainal, Sugeng Triwahyono, Adam F. Lee, Karen Wilson, Yun Hin Taufiq-Yap

**Affiliations:** 1 Catalysis Science and Technology Research Centre (PutraCat), Faculty of Science, Universiti Putra Malaysia, UPM, Serdang 43400, Selangor, Malaysia; Shanons1986@yahoo.com (M.A.); goh_chee_keong@rp.edu.sg (C.K.G.); zulkar@upm.edu.my (Z.Z.); 2 School of Applied Science, Republic Polytechnic, 9 Woodlands Ave 9, Singapore 738964, Singapore; 3 Department of Chemistry, Faculty of Science, Universiti Teknologi Malaysia, UTM, Johor Bahru 81310, Johor, Malaysia; sugeng@utm.my; 4 Centre for Applied Materials & Industrial Chemistry (CAMIC), School of Science, RMIT University, 124 La Trobe Street, Melbourne, VIC 3000, Australia; adam.lee2@rmit.edu.au; 5 Faculty of Science and Natural Resources, Universiti Malaysia Sabah,Kota Kinabalu 88300, Sabah, Malaysia

**Keywords:** hierarchical zeolites, HZSM-5, solvothermal, fatty acids, deoxygenation, biofuels

## Abstract

Solid acid catalyzed cracking of waste oil-derived fatty acids is an attractive route to hydrocarbon fuels. HZSM-5 is an effective acid catalyst for fatty acid cracking; however, its microporous nature is susceptible to rapid deactivation by coking. We report the synthesis and application of hierarchical HZSM-5 (h-HZSM-5) in which silanization of pre-crystallized zeolite seeds is employed to introduce mesoporosity during the aggregation of growing crystallites. The resulting h-HZSM-5 comprises a disordered array of fused 10–20 nm crystallites and mesopores with a mean diameter of 13 nm, which maintain the high surface area and acidity of a conventional HZSM-5. Mesopores increase the yield of diesel range hydrocarbons obtained from oleic acid deoxygenation from ~20% to 65%, attributed to improved acid site accessibility within the hierarchical network.

## 1. Introduction

The synthesis of renewable fuels from triglycerides and fatty acids, derived from non-food plant oils, low grade waste cooking oils [1], or algal oils [2], is an attractive means to replace fossil energy resources [3,4,5,6]. Shape selective zeolite catalysts have been widely used for catalytic cracking, with HZSM-5 the most efficient for fatty acid conversion to paraffins, olefins, and aromatic compounds in the range of gasoline and kerosene fractions [7]. However, the monomodal, micropore network of this zeolite hinders the conversion of bulky molecules [8] and renders the catalyst susceptible to pore blockage and concomitant deactivation [9,10].

Synthesis of hierarchical zeolites with a secondary mesopore network is an attractive approach to overcome intraparticle diffusion limitations in microporous zeolites [11,12,13,14]. Top-down or bottom-up methods to introduce pore hierarchy involve the introduction of mesopores by respective post dealumination/desilication of pre-formed zeolites or incorporation of structure directing agents during zeolite nanocrystal assembly [15]. Regarding the latter, assembly of zeolite nanocrystals into hierarchically porous aggregates is reported for Mordenite Framework Inverted (MFI) [16,17,18], Beta polymorph A (BEA) [17,19], and Mordenite (MOR) [20] via surface passivation by silanizing agents which hinder aggregation of zeolitic seeds during their hydrothermal aging and serve as soft mesopore templates. In such approaches, hierarchical nanozeolite assemblies of 200–400 nm diameter are formed from 5–10 nm zeolite nanocrystals wherein the overall morphology depends on the concentration of surface silanizing agent and hydrothermal processing conditions [16,17,18,21]. Solvothermal approaches employing mixed organic solvents (e.g., toluene and alcohols) are also reported which offer additional control over crystal growth to produce uniform 20–50 nm crystallites [22,23,24,25]. However, the use of auxiliary solvents in chemical synthesis is undesirable from a Green Chemistry perspective and an alternative, safer and more economically viable zeolite precursor to the widely adopted aluminium isopropoxide is desirable. Although Al_2_(SO_4_)_3_ has been employed as an aluminium source in ZSM-5 synthesis [26,27], there are no reports of its use in the bottom-up synthesis of hierarchical zeolites. By using aluminium sulfate and tetrapropyl ammonium hydroxide, a simplified synthesis is enabled, which produces ZSM-5 as the ammonium form, and avoids the need for repeated ion-exchange treatments encountered when alkaline bases are employed [28].

**Scheme 1:**
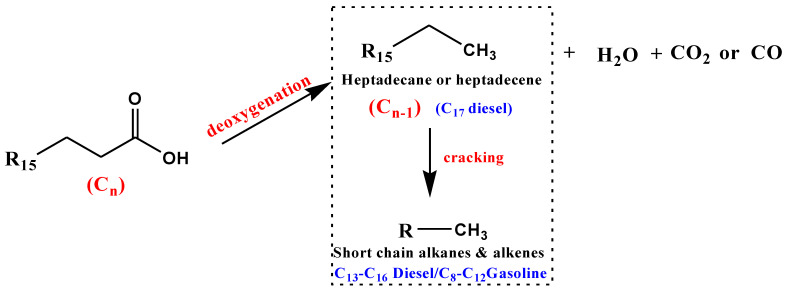
General route for deoxygenation of fatty acids by decarboxylation and decarbonylation.

Fatty acids and their derivatives can be deoxygenated using zeolite catalysts to produce fuel-like hydrocarbons via decarboxylation/decarbonylation process which liberate CO, CO_2_, and H_2_O as by-products (Scheme 1) [29,30,31,32].

The impact of pore hierarchy in nanosized zeolite Y with crystal sizes spanning 20–2700 nm has been explored for triolein deoxygenation [33], wherein decreased crystallite sizes increased the extent of intercrystallite mesoporosity. Such enhanced mesporosity enhances triolein conversion to deoxygenated liquid products, with both increased hydrocarbon yield and selectivity for diesel ranged hydrocarbons (and suppressed heavy paraffin formation). The high activity of hierarchical zeolites reflects improved accessibility of triolein to the external surface of zeolite nanocrystals [33]. Likewise, the introduction of mesopores in ZSM-5 significantly enhances the catalytic cracking of oleic acid to C2–C4 olefins and aromatics at 500 °C, with a maximum selectivity of 38% for light olefins. Mesoporous HZSM-5 had a greater external surface area and mesopore volume than conventional HZSM-5, facilitating reactant diffusion to Brönsted acid sites [34]. However, to date, hierarchically porous ZSM-5 has not been applied for the deoxygenation of oleic acid to green diesel.

Further improvements to the textural properties of hierarchical nanozeolites necessitate a more efficient and economical route to their synthesis. Tetrapropylammonium bromide (TPABr) and [3-(trimethoxysilyl) propyl] octadecyldimethylammonium chloride (TPAOC) templates used in previous work [34] are quite expensive. Moreover, mesoporous zeolites produced using the amphiphilic organic surfactants cetyltrimethylammonium bromide (CTAB) and TPAOC exhibit much poorer structural stability and weaker acidity than conventional zeolites. Herein, we report the synthesis of hierarchical nanozeolite ZSM-5 with a high external surface area and acidity for the catalytic cracking of oleic acid using hexadecyltrimethoxysilane (HDTM) as an inexpensive surface silanising agent.

## 2. Materials and Methods

### 2.1. Zeolite Synthesis

Hierarchical HZSM-5 (h-HSZM-5) was synthesized adapting the method of Vuong et al. [24] but without the addition of auxiliary solvent. Hierarchical h-HZSM-5 and conventional c-HZSM-5 were synthesized from the same parent zeolite seed crystals having a molar ratio of 60 SiO_2_:1 Al_2_O_3_:32 tetrapropylammonium hydroxide (TPAOH). Zeolite seed crystals were first prepared by adding 3.35 g tetraethoxysilicate (98%, Sigma, St. Louis, MO, USA) to 6.50 g TPAOH (20% in water, Sigma, St. Louis, MO, USA), stirring for 15 min, and then adding 0.22 g aluminium sulfate hexadecahydrate (Al_2_(SO_4_)_3_·16H_2_O, ≥95%, Sigma, St. Louis, MO, USA). The resulting mixture was then aged for 24 h at 30 °C in a polypropylene bottle under stirrer. h-HZSM-5 was subsequently prepared by adding 1.08 g HDTM (≥85% Sigma, St. Louis, MO, USA), corresponding to 19 mol% with respect to the silica content in the gel, to the aged solution of zeolite seed crystals, and heating to 80 °C under stirring for 12 h to initiate the surface derivatization. The resulting solution was then transferred to a Teflon-lined, stainless steel static autoclave for hydrothermal processing in an oven at 150 °C for 5 days. The resulting crystalline products were recovered by centrifugation, washed with deionized water, and then dried overnight at 80 °C. Conventional zeolite c-HZSM-5 was prepared by hydrothermal processing of the zeolite seed crystals without HDTM addition. These materials were compared to a commercial HZSM-5 (Alfa Aesar, Ward Hill, MA, USA) with Si:Al = 15. The synthetic zeolites were calcined in air at 550 °C (1 °C/min ramp for 5 h) to remove the organic residues, and subsequently converted to protonated form (HZSM-5) by treatment with 1.0 M NH_4_NO_3_ (Sigma, St. Louis, MO, USA) solution at 80 °C for 3 h, washing with deionised water, oven drying, and a second calcination at 550 °C (3 °C·min^−1^ ramp for 4 h). The calcined zeolites were stored in a desiccator prior to use.

### 2.2. Characterization

X-ray diffraction (XRD) analysis was performed using a Shimadzu XRD-6000 (Shimadzu Corporation, Kyoto, Japan) with Cu K_α_ radiation (λ = 0.154 nm) and a scan rate of 2.0° min^−1^. Crystallinity of the synthesized zeolite samples was determined from the relative intensity of the most intense (501) reflection at 2θ = 23.1° in the uncalcined conventional versus hierarchical zeolites (extensively washed to remove excess template and oven dried at 100 °C prior to analysis). Infrared spectra were recorded at 4 cm^−1^ resolution using a Perkin Elmer Fourier Transform Infrared (FTIR) spectrometer model 100 (PerkinElmer, Inc., Waltham, MA, USA) equipped with an attenuated total reflectance (ATR-IR) accessory. The zeolite Si:Al ratios were determined by inductively coupled plasma-optical emission spectroscopy (ICP-OES) on a Thermo Scientific iCAP 7000 instrument (Thermo Fisher Scientific, Waltham, MA, USA) equipped with an ASX-520 autosampler; samples were digested using an aqueous mixture of 2:1:1 HF/HNO_3_/HCl.

Sample morphology was determined by field emission scanning electron microscope (FESEM) on a JEOL model JSM-7600F (JEOL, Ltd., Tokyo, Japan) at an accelerating voltage of 10.00 kV equipped with energy-dispersive X-ray (EDX) spectroscopy (model Oxford Instruments, X-Max, Abingdon, Oxfordshire). The sample was dispersed on silver adhesive paint atop a metal stub and coated with a thin layer of palladium using a BIO-RAS, Sputter Coater. Transmission electron microscope (HR-TEM) images were recorded using a Hitachi H-7100 microscope (Hitachi, Ltd., Tokyo, Japan) at 200 kV accelerating voltage. Thermogravimetric analysis (TGA) was performed using a Mettler Toledo TGA/SDTA 851 instrument (Mettler Toledo, Columbus, OH, USA) under 30 mL·min^−1^ flowing N_2_ at 10 °C·min^−1^ ramp rate. Nitrogen porosimetry was performed using a Thermo Fisher Scientific instrument (Thermo Fisher Scientific, Waltham, MA, USA) at −196 °C. Samples were degassed at 250 °C for >12 h at 10^−3^ Torr. Specific surface areas were determined using the Brunauer–Emmett-Teller (BET) method on the adsorption branch of the isotherm between P/Po = 0.05–0.15, with information on micropores obtained using the t-plot method. Mean pore diameter and mesopore volume were estimated using the Barrett–Joyner–Halenda (BJH) method applied to the desorption isotherm for P/Po = 0.1–0.99.

Acidity was determined from the temperature programmed decomposition of sec-butylamine to butene and ammonia [35], which was quantified by TGA using a Mettler Toledo TGA/SDTA 851 (Mettler Toledo, Columbus, OH, USA). About 10 mg of sample was wetted with 1 mL sec-butylamine and air dried prior to drying overnight in an oven at 70 °C. The amine impregnated sample was then heated at 10 °C·min^−1^ from 50 to 820 °C under 50 mL·min^−1^ flowing N_2_. Acid site loadings were determined from the mass loss over the range 280–500 °C associated with evolved 2-butene from decomposition of the chemisorbed sec-butylamine.

Solid-state one-dimensional nuclear magnetic resonance (1D NMR) experiments were conducted at room temperature using a 11.7 T magnetic field on a high-resolution Bruker AVANCE III HD 400 spectrometer (Bruker Corporation, Billerica, MA, USA) with a magic-angle spinning (MAS) probe. Powder samples were tightly packed in conical Andrew-type 4 mm hollow rotors. Solid state ^13^C NMR experiments were conducted at 100.63 MHz and chemical shifts referenced to the corresponding nuclei in tetramethylsilane. ^13^C cross-polarisation (CP) MAS NMR spectra were recorded with TOSS spinning sideband suppression with a 1 s recycle delay, 3 ms contact time, and 3600 transients.

### 2.3. Catalytic Deoxygenation

Oleic acid deoxygenation was performed at 360 °C in a 250 mL stainless steel semi-batch reactor (Figure S1), using 5 g of oleic acid and 0.5 g of zeolite. The reactor was continuously purged with 30 mL·min^−1^ flowing N_2_ to remove gaseous CO and CO_2_ products; evolved hydrocarbon products were condensed in a close-coupled vapour trap held at 16 °C. Reactions were run for 1 h, with liquid products analysed by gas chromatography (GC) using an Agilent Technology 7890 GC (Agilent Technologies, Inc., Santa Clara, CA, USA) equipped with flame ionisation detector and a non-polar HP-5 capillary column (30 m × 0.32 mm × 0.25 μm). The conversion of oleic acid, yield of hydrocarbons, and diesel selectivity were determined as follows:

$$Conversion\;\left(wt.\;\%\right)=\;\frac{Oleic\;acid\;\left(initial\;mol-\;final\;mol\right)}{Oleic\;acid\;\left(initial\;mol\right)}\;\times 100\%\;\;\;\;\;\;\;\;\;\;\;\;$$

$$Hydrocarbon\;yield\;\;\left(\%\right)=\;\frac{\mathrm{experimental}\;\mathrm{concentration}\;\mathrm{of}\;C_{8}-C_{18}\;\mathrm{hydrocarbons}\;}{\mathrm{theoretical}\;\mathrm{concentration}\;\mathrm{of}\;C_{8}-C_{18}\;\mathrm{hydrocarbons}}\times 100\%\;$$

$$Selectivity\;of\;product\;\%=\frac{\mathrm{sum}\;\mathrm{of}\;\mathrm{concentration}\;\mathrm{of}\;\mathrm{selected}\;\mathrm{hydrocarbons}\;}{\;\mathrm{total}\;\mathrm{concentration}\;\mathrm{of}\;\mathrm{products}}\;\;\times 100\%\;\;\;\;\;\;\;\;\;\;$$

## 3. Results

### 3.1. Materials Characterisation

**Figure 1:**
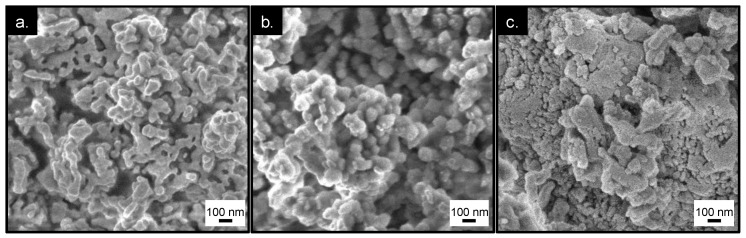
Field emission scanning electron microscope (FESEM) images of uncalcined (**a**) h-HZSM-5, (**b**) c-HZSM-5 and (**c**) HZSM-5.

The hydrothermal synthesis of HZSM-5 and influence of hexadecyltrimethoxysilane (HDTM) on nanocrystal aggregation was first investigated for uncalcined materials. FESEM imaging (Figure 1a–c) revealed a “cauliflower-like” morphology for as-synthesized h-HZSM-5 and c-HZSM-5, comprising fused primary nanocrystals [36]. In contrast, commercial HZSM-5 comprised large irregular crystallites with a wide particle size distribution [37].

**Figure 2:**
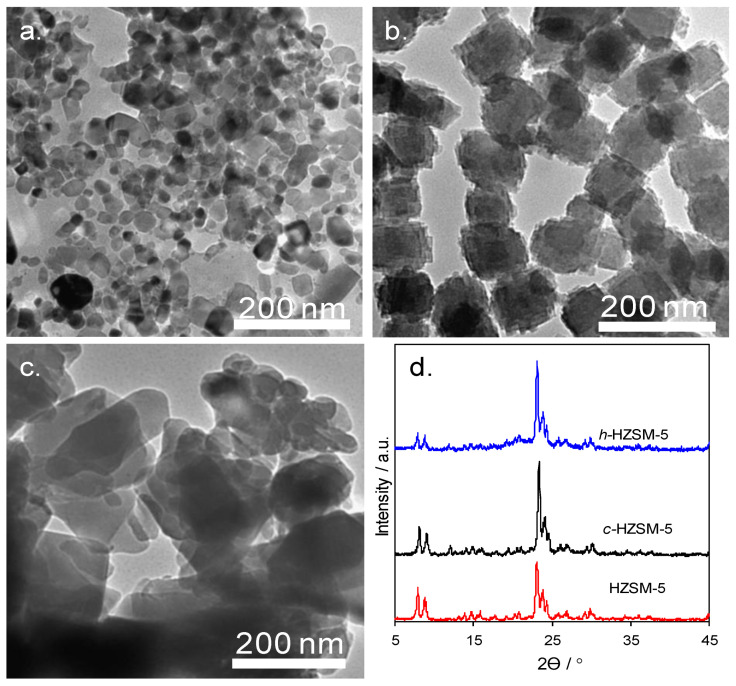
HR-TEMimages of uncalcined (**a**) h-HZSM-5, (**b**) c-HZSM-5, and (**c**) HZSM-5, and (**d**) powder *x*-ray diffraction (XRD) patterns for the three zeolites.

Corresponding TEM images (Figure 2a–c) further evidenced the impact of silanization on the aggregation of primary zeolite nanoparticles during hydrothermal processing. h-HZSM-5 comprised a disordered array of 20–50 nm fused nanocrystallites, whereas c-HZSM-5 exhibited larger cubic aggregates (90–130 nm). Powder XRD patterns (Figure 2d) revealed an Mordenite Framework Inverted (MFI) structure characteristic of the commercial zeolite; Scherrer analysis of reflections indicates similar volume averaged crystallite sizes for h-HZSM-5 and c-HZSM-5 of 24 and 27 nm respectively (Table 1). These observations suggest that HDTM influences the aggregation of primary zeolitic nanocrystals, but not their framework structure or size (which is determined by the initial gel composition and aging condition during the pre-crystallization step) [18].

Elemental analysis (Table 1) shows that the Si:Al ratio of uncalcined h-HZSM-5 is slightly higher than that of c-ZMS-5, attributed to extra Si arising from the HDTM silanization, and consistent with the increased carbon content of the derivatized zeolite. For c-HZSM-5 and HZSM-5, residual carbon mainly arises from the tetrapropylammonium (TPA) cationic template [38,39].

**Figure 3:**
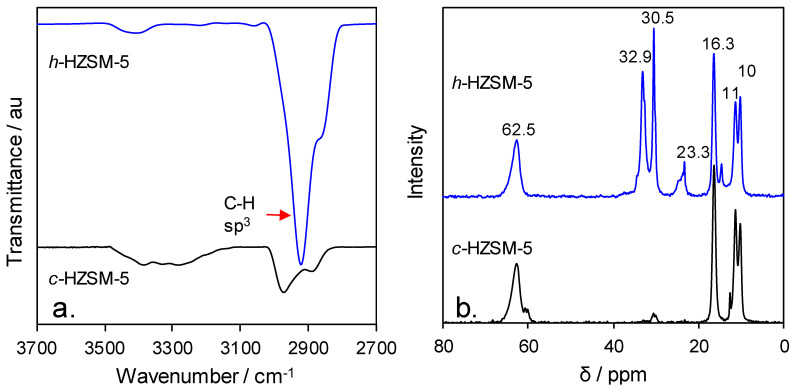
(**a**) ATR-IR) and (**b**) ^13^C NMR spectra of uncalcined h-HZSM-5 and c-HZSM-5.

Incorporation of surface passivating agents was also studied by FTIR and ^13^C NMR (Figure 3a,b). As-synthesized c-HZSM-5 exhibited a weak, broad IR band at 3100–3550 cm^−1^, attributed to hydrogen bonded surface Si–OH groups [40], and sharper, stronger bands at 2881 and 2950 cm^−1^ associated with the sp^3^ C–H stretch of TPA alkyl chain residues. h-HZSM-5 was dominated by intense IR bands at 2856 and 2919 cm^−1^ characteristic of sp^3^ C–H stretching modes of HDTM, indicating successful surface derivatization. ^13^C CP-MAS NMR spectra of c-HZSM-5 revealed peaks at 10.3, 11.3, 16.4, and 63 ppm associated with the TPA cationic template [18], with h-HZSM-5 exhibiting additional, strong peaks at 14.5, 23.5, 30.5, and 32.9 ppm due to the incorporation of HDTM.

**Figure 4:**
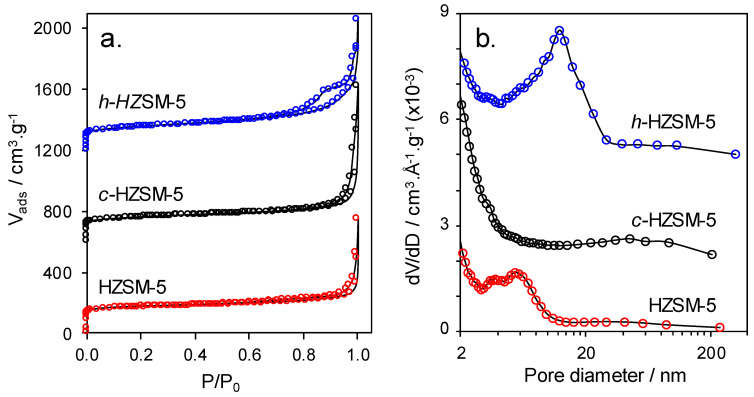
(**a**) N_2_ adsorption-desorption isotherms of calcined h-HZSM-5, c-HZSM-5, and HZSM-5, and (**b**) corresponding BJH pore size distributions.

Textural properties of the calcined zeolites were subsequently determined by N_2_ porosimetry (Figure 4). Adsorption-desorption isotherms of calcined h-HZSM-5 (Figure 4a) were intermediate between type II or IV, with an inflection point starting around P/P_0_ = 0.8, and hysteresis in the desorption branch associated with interparticle mesopore voids formed during directed aggregation of silanized particles (absent in commercial HZM-5 and c-HZSM-5) [25]. All samples exhibited similar BET surface areas (Table 2), comparable to those reported for hierarchical nanozeolites [25], although the h-HZSM-5 exhibited the least microporosity and a significantly higher mesopore volume than the commercial zeolite [41].

Pore size distributions evidenced well-defined mesopores of ~15 nm diameter for h-HZSM-5, whereas c-HZSM-5 exhibited negligible mesoporosity and the commercial zeolite only a small proportion of very small mesopores. HZSM-5 exhibited a small number of 4 nm pores owing to interparticle aggregation of zeolite crystals [22]. The h-ZSM-5 material prepared in this work exhibits a higher surface area and almost double the pore size and acid site loading of previously reported mesoporous ZSM-5 [34], suggesting that our surface silanization method avoids undesired surface passivation.

**Figure 5:**
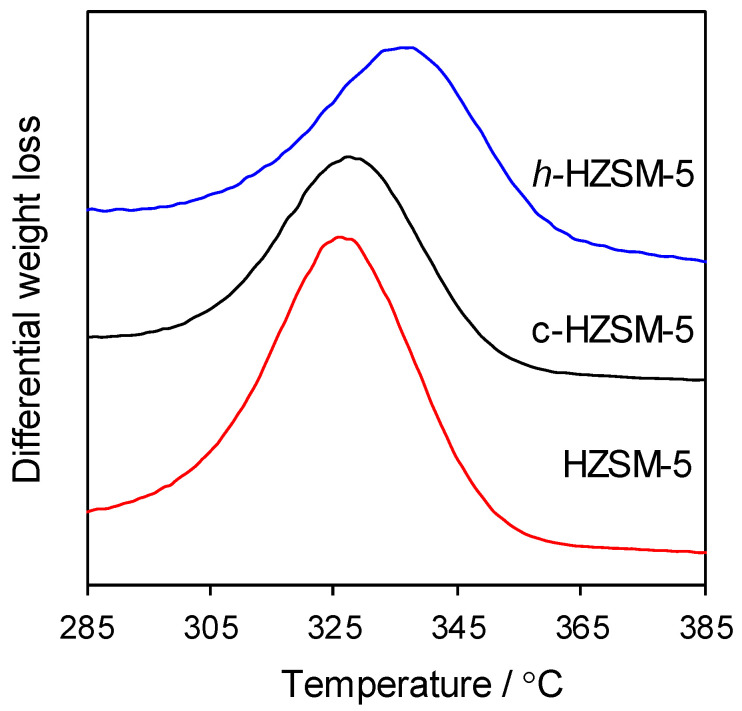
Differential weight loss from sec-butylamine decomposition to butene and NH_3_ over h-HZSM-5, c-HZSM-5, and HZSM-5.

Acidity was characterized by TGA of chemisorbed sec-butylamine, whose decomposition liberates butene (Figure 5) and ammonia, where increased acid strength leads to a decreased decomposition temperature [42]. Reactively-formed butene desorbed with peak maxima of 329 °C for commercial HZSM-5 and c-HZSM-5, consistent with similar, moderate strength acid sites. A slight increase in the butene peak maximum (to 339 °C) observed for h-HZSM-5 indicates slightly weaker acidity, a possible consequence of additional Si incorporated during HDTM silanization [21]. However, h-HZSM-5 exhibited the highest acid loading, which may reflect improved acid site accessibility by the bulky amine probe molecule to the internal mesopore network of the hierarchical structure.

### 3.2. Catalytic Deoxygenation of Oleic Acid

The activity of h-HZSM-5 for the solventless deoxygenation of oleic acid was subsequently compared against that of c-HZSM-5 and commercial HZSM-5. All zeolites (and a blank reaction without any catalyst) resulted in >99% oleic acid conversion at 300 °C and some residual char; for the blank reaction this reflected thermal cracking of oleic acid into short-chain carboxylic acids. Major products over the zeolites were linear and iso-alkanes/alkenes, with only trace alcohols, aromatics, cycloalkanes, and aldehydes observed (Table S1). Note that gaseous products were not analysed. The main aromatic product was dodecylbenzene, albeit at yields <3% in all cases. Hydrocarbon yield and selectivity are summarized in Table 3 and Figure S2; h-HZSM-5 exhibits a 3-fold greater hydrocarbon yield versus c-HZSM-5 and commercial HZSM-5, which is attributed to the enhanced accessibility of acid sites in the hierarchical zeolite to the fatty acid reactant [18,22,25]. By way of comparison [7], hydrothermal cracking of palmitic acid at 400 °C over ZSM-5 yielded mainly aromatics (notably xylene and toluene) and aliphatics (2-methyl-pentane and heptane) indicative of cracking, cyclisation, and isomerisation. The cracking of waste sunflower oil over ZSM-5 at 450 °C in a fixed bed process yielded 59 wt% hydrocarbons [43], and a core shell AlMCM-41@ZSM-5 composite yielded 39% of a bioliquid fuel comprising 47% and 36% green diesel and green gasoline respectively from Jatropha oil cracking at 400 °C [44].

## 4. Conclusions

Surface silanization of nanozeolite seeds with HDTM prior to hydrothermal aging directs mesopore formation and the formation of a hierarchical ZSM-5 (h-HZSM-5) catalyst. Nanozeolite seeds were themselves successfully synthesised from a low cost and readily available aluminium sulfate precursor, without auxiliary solvents, offering an environmentally benign route to h-HZSM-5 possessing large (15 nm) mesopores and a high surface area (570 m^2^g^−1^) comparable to commercial ZSM-5. The introduction of mesopores promotes enhanced access of bulky lipids to acid sites, promoting deoxygenation within the hierarchical zeolite framework to long-chain hydrocarbons (65% versus 20–25% for conventional HZSM-5) over competing cracking and char formation.

## Figures and Tables

**Table 1:** Physical properties of zeolite HZSM-5 materials following hydrothermal syntheses in the presence or absence of HDTM.

Sample	Si:Al Atomic Ratio ^a^	C:Si Atomic Ratio ^b^	Crystallite Size/nm ^c^
h-HZSM-5	20	5.3	24
c-HZSM-5	17	1.5	27
HZSM-5	15	1.9	19

^a^ Si:Al ratio from inductively coupled plasma-optical emission spectroscopy (ICP-OES); ^b^ energy-dispersive X-ray (EDX); ^c^ XRD.

**Table 2:** Textural properties of calcined zeolite ZSM-5 materials.

Sample	S*_BET_^a^ /m^2^g^−1^	S_MIC_^b^ /m^2^g^−1^	S_MES_^c^ /m^2^g^−1^	V_MIC_^b^ /cm^3^g^−1^	V_MES_^c^ /cm^3^g^−1^	V_TOT_^d^ /cm^3^g^−1^	Total Acid Site Loading ^e^ /mmol·g^−1^
h-HZSM-5	750	330	259	0.14	0.78	1.03	1.2 (0.002)
c-HZSM-5	595	444	173	0.19	0.59	1.26	0.7 (0.001)
HZSM-5	643	518	132	0.22	0.49	0.52	0.8 (0.001)

^a^ Brunauer–Emmett-Teller (BET) surface area; ^b^ S_MIC_ and V_MIC_ from t-plot analysis; ^c^ S_Mes_ and V_MES_ from BJH analysis; ^d^ V_TOT_ determined at P/Po = 0.99; ^e^ Mass loss between 280–500 °C (density in mmol·m^−2^ shown in parentheses).

**Table 3:** Yield and product selectivity for the catalytic cracking of oleic acid ^a^.

Catalyst ^a^	Hydrocarbon Yield /%	Productivity ^b^ /mmol min^−1^ g^−1^	Alkene/Alkane Product Selectivity /%		Mass Balance ^c^ /%
			C_8_–C_12_	C_13_–C_17_	
h-HZSM-5	65	0.70	22	78	80
c-HZSM-5	24	0.26	28	72	78
HZSM-5	20	0.21	22	78	83
Blank	4	-	13	87	-

^a^ 300 °C for 1 h; all samples gave ~99% oleic acid conversion; ^b^ based on 1 h alkane and alkene yields; ^c^ mass balance based on total mass of liquid product and remaining residue (without gas composition).

## Data Availability

Not applicable.

## Supplementary Materials

The following are available online at https://www.mdpi.com/2079-4991/11/3/747/s1, Figure S1: Schematic diagram of semi-batch DO reactor, Figure S2: Distribution of alkanes and alkenes in DO products, Table S1: Overall product distribution in DO reactions.
