# Supplementary material for: Hierarchical HZSM-5 for Catalytic Cracking of Oleic Acid to Biofuels
Source: Nanomaterials (Basel). 2021 Mar 16;11(3):747. doi: 10.3390/nano11030747 (PMC8002341; doi:10.3390/nano11030747)
Supplement: Supplementary file 1 [file nanomaterials-11-00747-s001.pdf]

nanomaterials

Supplementary Materials

Hierarchical HZSM-5 for Catalytic Cracking of Oleic Acid to Biofuels

Mahashanon Arumugam [1], Chee Keong Goh [1,2], Zulkarnain Zainal [1], Sugeng Triwahyono [3], Adam F. Lee [4], Karen Wilson [4,*] and Yun Hin Taufiq-Yap [1,*]

[1] Catalysis Science and Technology Research Centre (PutraCat), Faculty of Science, Universiti Putra Malaysia, UPM Serdang, Selangor 43400, Malaysia; Shanons1986@yahoo.com (M.A.); goh_chee_keong@rp.edu.sg (C.K.G.); zulkar@upm.edu.my (Z.Z.)
[2] School of Applied Science, Republic Polytechnic, 9 Woodlands Ave 9, 738964, Singapore
[3] Department of Chemistry, Faculty of Science, Universiti Teknologi Malaysia, UTM Johor Bahru, Johor, 81310, Malaysia; sugeng@utm.my
[4] Centre for Applied Materials & Industrial Chemistry (CAMIC), School of Science, RMIT University, 124 La Trobe Street, Melbourne, VIC 3000, Australia; adam.lee2@rmit.edu.au
* Correspondence: karen.wilson2@rmit.edu.au (K.W.); taufiq@upm.edu.my (Y.H.T.-Y.); Tel.: +61-(03)-9925-2122 (K.W.); +603-7967-6954 (Y.H.T.-Y.)

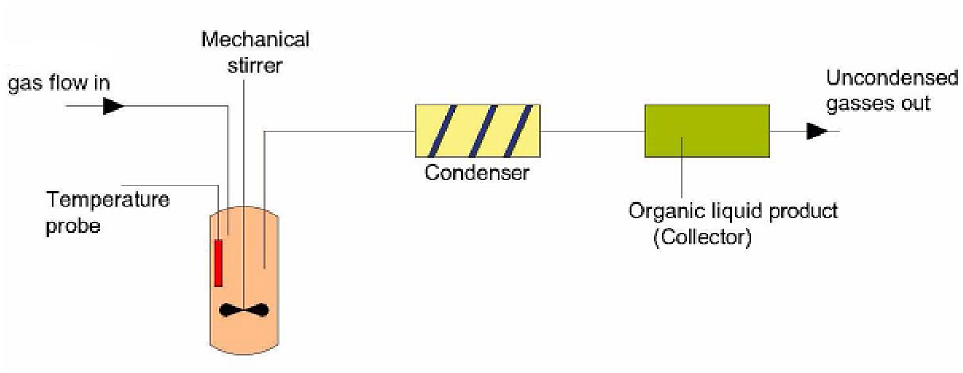

Figure S1. Schematic diagram of semi-batch DO reactor.

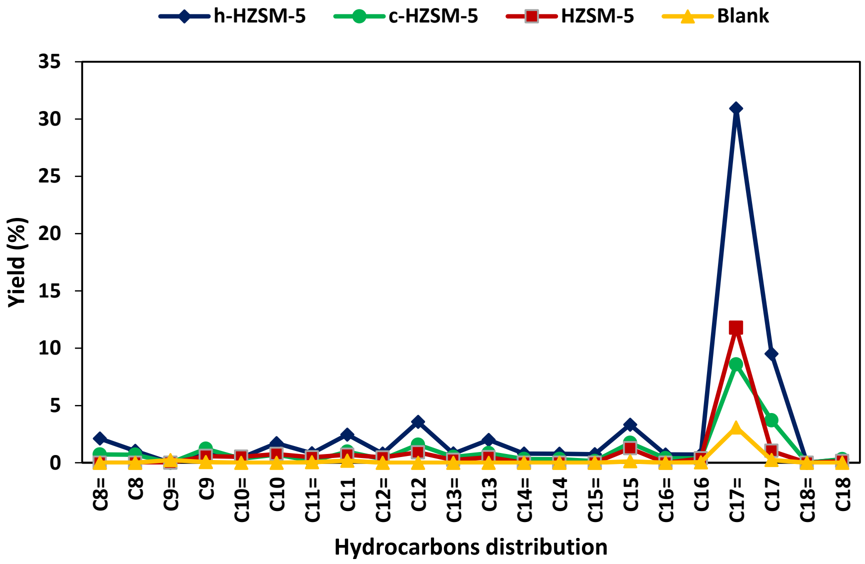

Figure S2. Distribution of alkanes and alkenes in DO products.

Table S1. Overall product distribution in DO reactions.

	Area %		
	**h-HZSM-5**	**c-HZSM-5**	**HZSM-5**
**Hydrocarbons (C8–C20)**	73	80.8	78.6
**Heavy hydrocarbons (>C20)**	0	0	1.3
**Cyclic hydrocarbon**	11	6.1	10.5
**Alcohols**	8	6.4	5
**Aldehydes**	0	0.4	1.3
**Ketones**	3	1.3	1.4
**Aromatic**	3	3.8	1.9
**Others**	2	0.4	0
